# Quantile-Dependent Expressivity of Serum Uric Acid Concentrations

**DOI:** 10.1155/2021/3889278

**Published:** 2021-09-02

**Authors:** Paul T. Williams

**Affiliations:** Lawrence Berkeley National Laboratory, Molecular Biophysics & Integrated Bioimaging Division, 1 Cyclotron Road, Berkeley, CA 94720, USA

## Abstract

**Objective:**

“Quantile-dependent expressivity” occurs when the effect size of a genetic variant depends upon whether the phenotype (e.g., serum uric acid) is high or low relative to its distribution. Analyses were performed to test whether serum uric acid heritability is quantile-specific and whether this could explain some reported gene-environment interactions.

**Methods:**

Serum uric acid concentrations were analyzed from 2151 sibships and 12,068 offspring-parent pairs from the Framingham Heart Study. Quantile-specific heritability from offspring-parent regression slopes (*β*_OP_, *h*^2^ = 2*β*_OP_/(1 + *r*_spouse_)) and full-sib regression slopes (*β*_FS_, *h*^2^ = {(1 + 8*r*_spouse_*β*_FS_)^0.5^ − 1}/(2*r*_spouse_)) was robustly estimated by quantile regression with nonparametric significance assigned from 1000 bootstrap samples.

**Results:**

Quantile-specific *h*^2^ (±SE) increased with increasing percentiles of the offspring's sex- and age-adjusted uric acid distribution when estimated from *β*_OP_ (*P*_trend_ = 0.001): 0.34 ± 0.03 at the 10^th^, 0.36 ± 0.03 at the 25^th^, 0.41 ± 0.03 at the 50^th^, 0.46 ± 0.04 at the 75^th^, and 0.49 ± 0.05 at the 90^th^ percentile and when estimated from *β*_FS_ (*P*_trend_ = 0.006). This is consistent with the larger genetic effect size of (1) the *SLC2A9* rs11722228 polymorphism in gout patients vs. controls, (2) the *ABCG2* rs2231142 polymorphism in men vs. women, (3) the *SLC2A9* rs13113918 polymorphism in obese patients prior to bariatric surgery vs. two-year postsurgery following 29 kg weight loss, (4) the *ABCG2* rs6855911 polymorphism in obese vs. nonobese women, and (5) the *LRP2* rs2544390 polymorphism in heavier drinkers vs. abstainers. Quantile-dependent expressivity may also explain the larger genetic effect size of an *SLC2A9*/*PKD2*/*ABCG2* haplotype for high vs. low intakes of alcohol, chicken, or processed meats.

**Conclusions:**

Heritability of serum uric acid concentrations is quantile-specific.

## 1. Introduction

Serum uric acid concentrations reflect the equilibrium between renal clearance and endogenous uric acid produced from food-derived purines [1]. Hyperuricaemia, defined as uric acid > 404 or >417 *μ*mol/L (>6.8 or >7 mg/dL) [2], occurs when renal excretion is inadequate or uric acid is overproduced, for example, due to excessive intake of sugar-sweetened beverages and purine-rich foods [2]. Inadequate excretion is mainly due to the high reabsorption of filtered urate in the renal proximal tubules [3]. Hyperuricaemia can lead to gout, i.e., an inflammatory response within joints and tissues due to the deposition of urate crystals [2]. Age, male sex, obesity, alcohol consumption, and insulin resistance are also associated with increased hyperuricaemia and gout risk [2]. Hyperuricaemia is a risk factor for diabetes, hypertension, cardiovascular disease, and chronic kidney disease [2].

Individual variability in serum uric acid concentrations is known to be partially genetic, with heritability estimated from twin and family studies varying from 25% to 73% [4–9]. The 28 loci with genome-wide significance identified thus far account for about 7% of the interindividual variation in uric acid concentrations, of which two, glucose transporter type 9 (*SLC2A9*) and ATP-binding cassette subfamily G member 2 (*ABCG2*), account for about half of the genetic variance explained [10]. SLC2A9 in chromosome 4p16-15.3 encodes glucose transporter 9 (GLUT9) that reabsorbs uric acid in renal tubules [11]. *ABCG2* in chromosome 4q22 encodes ATP-binding cassette subfamily G member 2 (ABCG2) that reduces the transportation activity, resulting in hyperuricaemia [11]. A genome-wide association study of Japanese showed higher uric acid concentrations for the rs2544390 T-allele at intron 1 of*LRP2* at chromosome 2q24-31 that encodes low-density lipoprotein receptor-related protein 2 (megalin) [12]. How megalin affects urate metabolism is not currently known.

“Quantile-dependent expressivity” hypothesizes that the effects of genetic variants on phenotypes may depend on whether the phenotype (e.g., uric acid concentration) is high or low relative to its distribution [13]. The heritability of adiposity [13, 14]; plasma concentrations of triglyceride [13, 15], total cholesterol [16], high-density lipoproteins [13, 17, 18], leptin [19], adiponectin [20], plasminogen activator inhibitor type-1 [21], and C-reactive protein concentrations [22]; postprandial lipemia [23]; pulmonary function [24]; and intakes of alcohol [25] and coffee [26] are quantile-dependent, whereas height and the intakes of other macronutrients are not [13, 14, 25]. An important consequence of quantile-dependent expressivity is that the selection of subjects by characteristics that distinguish high vs. low phenotype values is expected to produce different genetic effects [18]. Traditionally, these have been interpreted as gene-environment interactions where environmental conditions modify genetic influences or where genotypes modify the susceptibility of the phenotype to the environment [18]. However, many reported gene-drug, gene-diet, and gene-environment interactions have been shown to be potentially attributable wholly or in part to quantile-dependent expressivity for adiposity (56 examples of interactions [14]), postprandial lipemia (64 examples [23]), serum triglycerides (76 examples [15]), total cholesterol (22 examples [16]), high-density lipoprotein cholesterol (88 examples [17, 18]), adiponectin (15 examples [20]), leptin (16 examples [19]), plasminogen activator inhibitor type-1 (21 examples [21]), and C-reactive protein concentrations (50 examples [22]).

Precision medicine attempts to identify genetic markers to identify patients who are most likely to benefit from medical treatment. When quantile-dependent expressivity changes the genetic effect size when the phenotype is increased or decreased, the genotype-specific changes in the phenotype cannot move in parallel [13–15]. In this case, the genetic marker may simply track the change in heritability associated with higher vis-à-vis lower phenotype values rather than revealing a physiological explanation for individual differences in treatment response.

It is not known whether uric acid heritability is quantile-dependent nor whether some of its gene-environment interactions may be attributable to quantile-dependent expressivity when subjects are selected for conditions that distinguish high vs. low phenotype values. Therefore, quantile regression [27, 28] was applied to the uric acid concentrations of sibships and offspring-parent pairs from the Framingham Heart Study [29–31] to estimate heritability in the narrow sense (*h*^2^ [32]) at different quantiles of the uric acid distribution. The discussion presents several purported interactions involving uric acid concentrations that might be more simply explained by quantile-dependent expressivity.

## 2. Methods

The Framingham Study data were obtained from the National Institutes of Health FRAMCOHORT, GEN3, FRAMOFFSPRING Research Materials obtained from the National Heart, Lung, and Blood Institute (NHLBI) Biologic Specimen and Data Repository Information Coordinating Center. The hypothesis tested was not considered a part of the initial Framingham Study design and is exploratory. Our analyses of these data were approved by the Lawrence Berkeley National Laboratory Human Subjects Committee (HSC) for protocol “Gene-environment interaction vs. quantile-dependent penetrance of established SNPs (107H021)” LBNL holds Office of Human Research Protections Federal wide Assurance number FWA 00006253, Approval number: 107H021-13MR20. All data collection was conducted under the direction of the Framingham Heart Study human use committee guidelines, with signed informed consent from all participants or parent and/or legal guardian if <18 years of age.

Uric acid concentrations were determined for examinations 1, 2, 3, 4, and 13 of the Original Framingham Heart Study Cohort, examinations 1 and 2 of the Offspring Cohort, and examination 1 of the Third Generation Cohort. Nonfasting serum uric acid concentrations from the Original Cohort were measured as described by Jacobson [33]. Fasting serum uric acid concentrations from the Offspring and Third Generation Cohorts were measured on an autoanalyzer using a phosphotungstic acid reagent [34]. Mean differences between cohorts due to fasting status and methodological procedures should have been eliminated by the calculation of age- and sex-adjusted residuals with each cohort. Results are presented as *μ*mol/L (1 mg/dL = 59.48 *μ*mol/L).

### 2.1. Statistics

The primary hypothesis is whether the urate heritability is quantile-dependent. The statistical methods employed have been described in detail [14–26]. Briefly, age and sex adjustment was performed using standard least-squares regression within each cohort separately with sex, age, age^2^, sex × age, and sex × age^2^ as independent variables. Individual subject uric acid concentrations were obtained by averaging adjusted concentrations over all available exams. Offspring-parent regression slopes (*β*_OP_) were computed using parents from the Original Cohort and their offspring who participated in the Offspring Cohort and using parents of the Offspring Cohort and their offspring who participated in the Third Generation Cohort. Sibships were identified from the Third Generation and Offspring Cohorts. Full-sibling regression slopes (*β*_FS_) were obtained by forming all *k*_*i*_(*k*_*i*_ − 1) sibpair combinations for the *k*_*i*_ siblings in sibship *i* and assigning equal weight to each sibling as previously described [35]. Simultaneous quantile regression was performed using the sqreg command of Stata (version. 11, StataCorp, College Station, TX), bootstrap resampling was used to estimate variances and covariance, and orthogonal polynomials were used to test for linear, quadratic, and cubic trends in the regression slopes between the 5^th^ and 95^th^ percentiles of the offspring or sib uric acid distribution [36]. Heritability in the narrow sense (*h*^2^) was calculated by *h*^2^ = 2*β*_OP_/(1 + *r*_spouse_), where *r*_spouse_ is the spouse correlation, and by *h*^2^ = {(1 + 8*β*_FS_*r*_spouse_)^0.5^ − 1}/2*r*_spouse_ under specific restrictive assumptions [32]. “Quantile-specific heritability” refers to the heritability statistic, whereas “quantile-dependent expressivity” refers to the biological phenomenon of the trait expression being quantile-dependent. Results are presented as the mean ± SE.

The results from several published studies were reinterpreted from the perspective of quantile-dependent expressivity. This was done using genotype-specific mean uric acid concentrations presented in the original articles or by extracting them from published graphs using the Microsoft PowerPoint formatting palette (Microsoft Corporation, Redmond, WA) as previously described [23]. The location of the SNPs is presented in Supplementary Table 1. Their interpretations are not necessarily those of the original articles.

## 3. Results

As expected, Table 1 shows that average uric acid concentrations were significantly higher in men than women (*P* < 10^−16^).

### 3.1. Traditional Estimates of Familial Concordance and Heritability

Spouse uric acid concentrations were significantly but weakly correlated (*r*_spouse_ = 0.1062). The offspring-parent regression slope (*β*_OP_ ± SE), calculated from 2318 offspring with one parent and 4875 offspring with two parents, was 0.2235 ± 0.0133, which corresponds to a heritability (*h*^2^) of 0.4041 ± 0.0240. Estimated *h*^2^ was similar in male and female offspring (0.4133 ± 0.0374 vs. 0.3956 ± 0.0307). The full-sib regression slope (*β*_FS_ ± SE : 0.2313 ± 0.0162) was calculated from 5761 full-sibs in 2151 sibships, which from Falconer's formula corresponds to heritability of *h*^2^ = 0.4419 ± 0.0322, with no significant male-female difference (0.4076 ± 0.0424 vs. 0.4714 ± 0.0355).

### 3.2. Quantile-Dependent Expressivity

**Figure 1:**
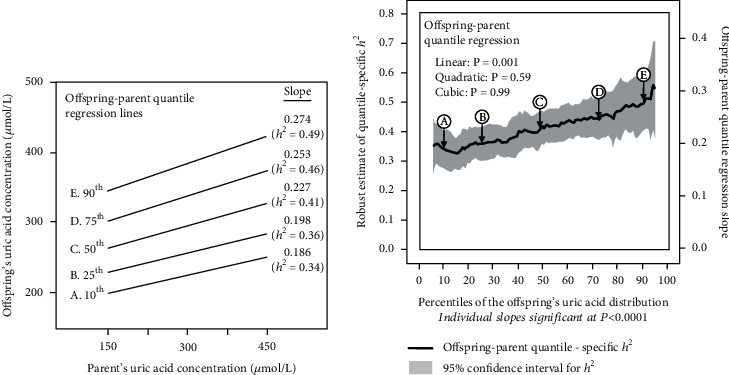
(a) Offspring-parent regression slopes (*β*_OP_) for selected quantiles of the offspring's uric concentrations from 12,068 offspring-parent pairs, with corresponding estimates of heritability (*h*^2^ = 2*β*_OP_/(1 + *r*_spouse_) [32], where the correlation between spouses was *r*_spouse_ = 0.1062. The slopes became progressively greater (i.e., steeper) with increasing quantiles of the uric acid distribution. (b) The selected quantile-specific regression slopes were included with those of other quantiles to create the quantile-specific heritability function in the lower panel. Significance of the linear, quadratic, and cubic trends and the 95% confidence intervals (shaded region) determined by 1000 bootstrap samples.

**Figure 2:**
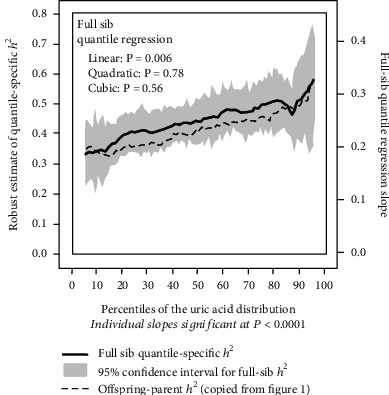
Quantile-specific full-sib regression slopes (*β*_FS_) from 5703 full-sibs in 2036 sibships, with corresponding estimates of heritability as calculated by *h*^2^ = {(8*r*_spouse_*β*_FS_ + 1)^0.5^ − 1}/(2*r*_spouse_) [32].

The offspring-parent regression slopes at the 10^th^, 25^th^, 50^th^, 75^th^, and 90^th^ percentiles of the offspring's uric acid distribution are presented in Figure 1(a), along with their corresponding heritability estimates. The regression slopes increased with increasing percentiles of the uric acid distribution. The heritability at the 90^th^ percentile was 47% greater than the heritability at the 10^th^ percentile (*h*^2^: 0.49 vs. 0.34, *P*_difference_ = 0.002). Figure 1(b) presents these slopes with those of the other percentiles between the 5^th^ and 95^th^ percentiles. It shows that heritability increased linearly (i.e., slope ± SE : 0.0020 ± 0.0005 for each percent increment, *P*_linear_ = 0.001) with increasing percentiles of the offspring's distribution. There was no statistically significant evidence of nonlinearity (i.e., *P*_quadratic_ = 0.60; *P*_cubic_ = 0.99). Individually, the quantile-specific heritability estimates were significant (*P* ≤ 10^−10^) for all percentiles between the 5^th^ and 95^th^ percentiles of the offspring's distribution. If the heritabilities were constant over all quantiles as usually assumed, then the line segments would be parallel in Figure 1(a), and Figure 1(b) displays a flat line with a zero slope.  Figure 2 displays the quantile regression analysis for *h*^2^ estimated from full-sib regression slopes (*β*_FS_). The full-sib regression slope increased 0.0012 ± 0.0004, and heritability increased 0.0024 ± 0.0008 with each one-percent increase in the uric acid distribution (*P*_linear_ = 0.008).

### 3.3. Replication

Additional support for quantile-dependent expressivity was obtained by analyzing the offspring-parent and full-sib quantile regression in the first-generation (offspring of the Offspring Cohort and their Original Cohort parents) and second-generation (offspring of the Third Generation Cohort and their Offspring Cohort parents) family sets separately. Spouse correlations (*r*_spouse_) were 0.1335 for the Original Cohort and 0.0728 for the Offspring Cohort. Heritability increased with increasing percentiles of the offspring distribution when estimated from *β*_OP_ (0.0024 ± 0.0011, *P* = 0.008) and *β*_FS_ (0.0022 ± 0.0012, *P* = 0.05) from the 2nd-generation cohort, and when estimated from *β*_OP_ (0.0018 ± 0.0011, *P* = 0.09) and *β*_FS_ (0.0028 ± 0.0012, *P* = 0.02) in the 1st-generation cohort.

## 4. Discussion

Our analyses of offspring-parent and full-sib pairs from the Framingham Heart Study suggest that serum uric acid concentrations exhibit quantile-dependent expressivity. Specifically, whereas genetic analyses traditionally assume that effect size is constant throughout the phenotype distribution, our analysis showed that heritability at the 90^th^ percentile of the offspring distribution was 47% larger than that at the 10^th^ percentile when estimated from offspring-parent regression and 53% larger when estimated from the full-sib regression slope. The results were generally replicated in the first- and second-generation family sets (the slightly weaker results for the Original Cohort parents may be due to their nonfasting samples and different urate assay vis-à-vis their offspring).

Quantile-dependent expressivity may explain some of the purported gene-environment interactions involving uric acid. Specifically, under quantile-dependent expressivity, the selection of subjects by characteristics that distinguish high vs. low uric acid concentrations is expected to show different genetic effects [18]. These differences have been traditionally attributed to gene-environment interactions due to biological interaction between the gene product and environmental conditions. None consider the differences in average uric acid levels between environmental conditions as their explanation. Quantile-dependent expressivity may arise from concentration-dependent effects of the genetic mutations affecting uric acid production, reabsorption, or clearance. The reported examples to follow represent interactions that are consistent with quantile-dependent expressivity because they show a larger genetic effect size at a higher average serum concentration.

### 4.1. Gout

**Figure 3:**
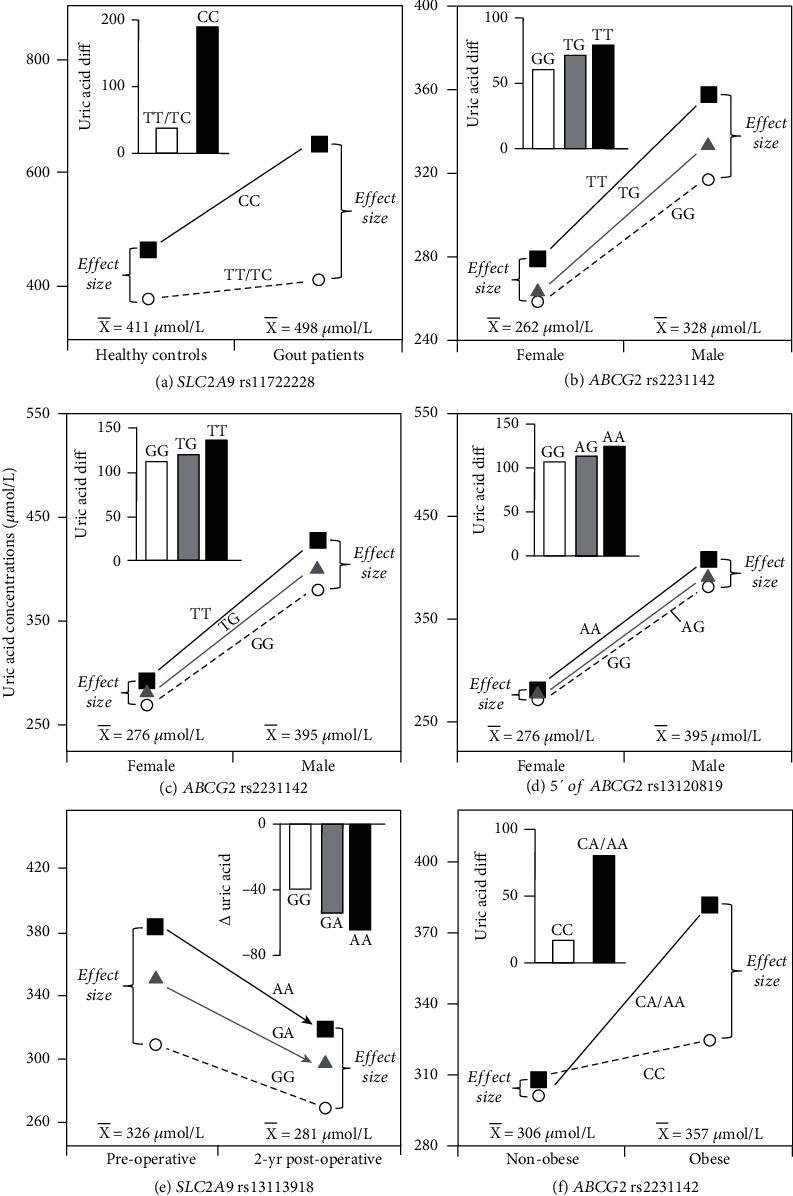
Precision medicine perspective of genotype-specific uric acid differences (histogram inserts) vs. quantile-dependent expressivity perspective (line graphs showing larger genetic effect size when average uric acid concentrations were high) for (a) Das Gupta et al.'s [37] 2018 report on the uric acid difference between gout patients and healthy controls by *SLC2A9* rs11722228 genotypes; (b) Yang et al.'s [42] 2014 report on the uric acid difference between males and females by *ABCG2* rs2231142 genotypes; (c) Lin et al.'s [43] report on the uric acid difference between males and females by *ABCG2* rs2231142 genotypes; (d) Lin et al.'s [43] 2020 report on the uric acid difference between males and females by rs13120819 genotypes located 5′ of *ABCG2*; (e) Sarzynski et al.'s [47] 2012 report on the uric acid difference before and after 29 kg weight loss following bariatric surgery by *SLC2A9* rs13113918 genotypes; (f) Cheng et al.'s [48] 2017 report on the uric acid difference between obese and nonobese women by *ABCG2* rs2231142 genotypes.

We are unaware of any published comparison of genetic effect size vs. mean uric acid concentrations. There is, however, Das Gupta et al.'s [37] report on uric acid levels in newly diagnosed male gout patients and controls. Gouty arthritis is the result of uric acid being crystallized as monosodium urate when serum concentrations exceed the normal range of 200–400 *μ*mol/L in men and 150–350 *μ*mol/L in women [2]. The histogram of Figure 3(a) examines whether the patient-control difference varied by rs11722228 genotypes of the *SLC2A9* gene that encodes the GLUT9 protein, the high-affinity uric acid transporter that is primarily responsible for uric acid reabsorption, and whose genetic variants explain about 3% of the variance in uric acid concentrations [38]. Their data show that an effect of gout on uric acid concentrations was significant for rs11722228 CC homozygotes (*P* = 0.03) but not carriers of the T-allele (*P* = 0.22).

The accompanying line graph in Figure 3(a) assesses whether the genotype differences were quantile-dependent, i.e., whether the genetic effect depended upon whether uric acid concentrations were high (patients) or low (controls). In fact, the line graph shows a greater difference between the CC homozygotes and T-allele carriers (241 ± 83 *μ*mol/L, *P* = 0.004) at the higher average concentrations represented by the gout patients (498 ± 33 *μ*mol/L) vis-à-vis the smaller genotype difference (88 ± 45 *μ*mol/L, *P* = 0.05) at the lower average concentrations of the healthy controls (411 ± 21 *μ*mol/L). This interpretation is consistent with the quantile-specific heritability of serum uric acid concentrations displayed in Figures 1 and 2.

### 4.2. Sex

The rs2231142 (Q141K) polymorphism produces a Glu141Lys amino acid substitution in exon 5 of *ABCG2* gene [39]. Multiple reports [10, 40, 41] show that the urate raising effect of the *ABCG2* rs2231142 gene polymorphism is greater in men than women. Köttgen et al.'s [10] GWAS of over 140,000 Europeans showed a 16.1 *μ*mol/L increase in men vs. 10.8 *μ*mol/L in women. In Han Chinese, Yang et al. [42] reported the significant interaction between sex and the *ABCG2* rs2231142 polymorphism (*P* = 0.02) as displayed in Figure 3(b). The male-female difference in uric acid concentrations was greatest in TT homozygotes, intermediate in TG heterozygotes, and least in GG homozygotes. The line graph shows that this could be attributed to the greater difference between genotypes at the higher average serum concentrations of the males than females. In another study of mostly Han Chinese ancestry, Lin et al. [43] reported significant sex by gene interaction for rs2231142 (*P*_interaction_ = 9.1 × 10^−9^) and rs13120819 located 5′ of *ABCG2* (*P*_interaction_ = 4.3 × 10^−7^) in subjects ≤ 50 years of age. The histograms of Figures 3(c) and 3(d) show the significant sex difference by genotype, which the line graph would attribute to the larger genetic effect for the higher mean uric acid concentrations of males than females. The authors attributed the difference to the attenuating effects of estrogen on the autosomal genetic effects, whereas quantile-dependent expressivity suggests that estrogen decreases uric acid concentrations and that the genetic effects are smaller at the lower serum concentrations.

### 4.3. Adiposity

The higher uric acid concentrations that are associated with greater visceral fat may be due to both increased production and poor excretion and clearance [44]. The greater influx of plasma free fatty acids into the hepatic portal vein and liver may stimulate hepatic triglyceride synthesis, which in turn promotes uric acid production [45, 46]. In addition, weight loss may improve renal uric acid clearance because hyperinsulinemia and insulin resistance are reduced.

Bariatric surgery reduces serum uric acid concentrations. Sarzynski et al. [47] reported that uric acid reductions following bariatric surgery were affected by *SLC2A9* rs13113918, a coding SNP that produces a synonymous substitution (Leu79Leu). Specifically, the histogram in Figure 3(e) (estimated from their figure 2) shows that the number of rs13113918 minor (A) alleles significantly affected two-year decreases in uric acid concentrations after 29 kg weight loss (*P*_interaction_ = 0.04). From the perspective of quantile-dependent expressivity, the line graph in Figure 3(e) shows that the large difference between genotypes before surgery, when average uric concentrations were high (326 *μ*mol/L), was substantially reduced two years postsurgery when average uric concentrations were less (281 *μ*mol/L).

Cross-sectionally, data presented by Cheng et al. [48] showed that the uric acid difference between obese and nonobese women was significantly greater in A-allele carriers than CC homozygote of the *ABCG2* rs2231142 polymorphism (Figure 3(f) histogram, *P*_interaction_ = 0.004). Alternatively, the line graph suggests a larger difference between genotypes at the higher mean concentrations of the obese vs. nonobese females. Another paper, by Brandstätter et al. [49], reported that BMI amplified the uric acid differences between genotypes of the *SLC2A9* rs6855911 (*P*_interaction_ = 0.035), rs7442295 (*P*_interaction_ = 0.023), rs6449213 (*P*_interaction_ = 0.024), and rs12510549 (*P*_interaction_ = 0.053) polymorphisms, consistent with quantile-dependent expressivity and the progressive increase in uric acid concentrations in going from a BMI of <30, to 30-40, and to >40 kg/m^2^ (cf.  Figure 1).

### 4.4. Alcohol Intake

**Figure 4:**
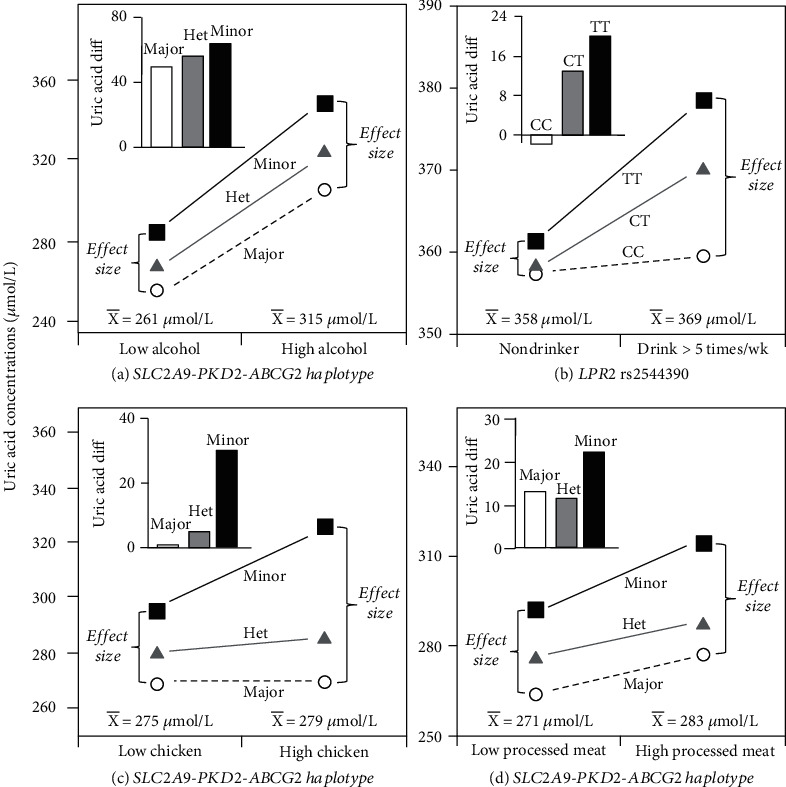
Precision medicine perspective of genotype-specific uric acid differences (histogram inserts) vs. quantile-dependent expressivity perspective (line graphs showing larger genetic effect size when average uric acid concentrations were high) for (a) Yang et al. [51] 2020 report on the uric acid difference due to drinking more vs. less than 10 g/d of alcohol by major, heterozygotic (het), and minor alleles of the *SLC2A9* rs3733591, *PKD2* rs2725220, and *ABCG2* rs2231142 haplotype; (b) Hamajima et al. [52] 2012 report on the uric acid difference between drinking >5 times per week vs. abstaining by the *LRP2* rs2544390 genotypes; (c) Yang et al. [51] 2020 report on the uric acid difference due to consuming more vs. less than 6.3 g/d of chicken by major, heterozygotic (het), and minor alleles of the *SLC2A9* rs3733591, *PKD2* rs2725220, and *ABCG2* rs2231142 haplotype; (d) Yang et al. [51] 2020 report on the uric acid difference due to consuming more vs. less than 3.0 g/d of processed meat by major, heterozygotic (het), and minor alleles of the *SLC2A9* rs3733591, *PKD2* rs2725220, and *ABCG2* rs2231142 haplotype.

Alcohol intake increases gout risk, particularly when consumed as beer and not as wine [50]. Alcohol affects renal urate transporters directly and uric acid excretion [3]. Yang et al. [51] reported that alcohol intake significantly modified the association between serum uric acid concentrations and a haplotype of *SLC2A9* rs3733591, *PKD2* rs2725220, and *ABCG2* rs2231142 in Korean adults (*P*_interaction_ = 0.002). They categorized the haplotype as major (0 minor alleles), heterozygote (1-2 minor alleles), and minor (3-4 minor alleles) alleles.  Figure 4(a) (derived from their figure 2) shows that the uric acid difference between Koreans consuming over 10 g/d of alcohol vs. less increased from being smallest for the major haplotype, intermediate for the heterozygote haplotype, the largest in the minor haplotype. Alternatively, the accompanying line graph attributes the histogram to the larger cross-sectional differences between haplotypes at the higher average uric acid concentrations of the heavier drinkers.

Hamajima et al. [52] reported that the gene encoding low-density lipoprotein receptor-related protein 2 (*LRP2*) in intron 1 (rs2544390) was significantly associated with uric acid concentrations in Japanese men (*P* = 0.01) and that the uric acid raising effect of the TT homozygotes was accentuated by alcohol intake (*P*_interaction_ = 0.005). The histogram in Figure 4(b) shows the greater difference between drinking >5 times/wk vs. abstinence in T-allele carriers vs. noncarriers, whereas the line graph emphasizes the greater genotype difference in the drinkers.

### 4.5. Diet

Higher intakes of red meat, seafood, and fructose-containing foods including soft drinks and low intakes of dairy products, caffeine, and vitamin C have been associated with gout and/or hyperuricaemia risk. Yang et al. [51] reported that consumption of chicken and processed meats significantly modified the association between serum uric acid concentrations and their aforementioned *SLC2A9*/*PKD2*/*ABCG2* haplotype (*P*_interaction_ = 0.003 and *P*_interaction_ = 0.007, respectively). The histograms in Figures 4(c) and 4(d) show that the uric acid difference between Koreans consuming over 6.3 g/d of chicken or 3.0 g/d of processed meat vs. less was greatest for the minor haplotype. Alternatively, the accompanying line graph shows the alternative interpretation where the cross-sectional differences between haplotypes were greatest at the higher average uric acid concentrations of the heavier consumers.

### 4.6. Limitations

Quantile-dependent expressivity is a novel concept, and for this reason, most articles do not provide the information needed to evaluate its applicability, namely, genotype-specific uric acid concentrations stratified by characteristics affecting overall average concentrations. Our reliance on the simple formula *h*^2^ = 2*β*_OP_/(1 + *r*_spouse_) and *h*^2^ = {(1 + 8*r*_spouse_*β*_FS_)^0.05^ − 1}/(2*r*_spouse_) to estimate heritability [32] is unlikely to embody the true complexity of uric acid inheritance. Our reinterpretations of the results presented by Das Gupta et al. [37], Yang et al. [42, 51], Lin et al. [43], Sarzynski et al. [47], Cheng et al. [48], Brandstätter et al. [49], and Hamajima and colleagues [52] do not disprove their original explanations, rather they suggest an alternative interpretation that warrants consideration.

In conclusion, quantile-dependent expressivity potentially provides a common principle underlying a plethora of published gene-drug and gene-environment interactions. The current analyses extend this phenomenon to uric acid concentrations. The gene-environment interactions cited above are examples potentially attributable to quantile-dependent expressivity.

## Figures and Tables

**Table 1:** Sample characteristics.

	Original Cohort	Offspring Cohort	Third Generation Cohort
Sample size			
Male	1257	2111	1854
Female	1312	2234	2098
Age^∗^ (years)			
Male	50.5 (7.9)	39.4 (10.4)	40.4 (8.8)
Female	50.0 (8.1)	38.8 (10.1)	40.1 (8.8)
BMI^∗^ (kg/m^2^)			
Male	26.3 (3.3)	26.7 (3.6)	28.0 (4.7)
Female	26.0 (4.5)	24.4 (4.7)	26.1 (6.1)
Uric acid^∗^ (*μ*mol/L)			
Male	306.8 (51.3)	376.5 (67.7)	375.6 (73.5)
Female	244.2 (49.9)	274.2 (61.4)	261.2 (62.2)

^∗^Mean (standard deviation).

## Data Availability

The data are not being published in accordance with the data use agreement between the NIH National Heart, Lung, and Blood Institute and Lawrence Berkeley National Laboratory. However, the data that support the findings of this study are available from the NIH National Heart, Lung, and Blood Institute Biologic Specimen and Data Repository Information Coordinating Center directly through the website https://biolincc.nhlbi.nih.gov/my/submitted/request/. Restrictions apply to the availability of these data, which were used under license for this study. Those wishing a copy of the data set should contact the Blood Institute Biologic Specimen and Data Repository Information Coordinating Center at the above website, where they can find information on human use approval and data use agreement requiring signature by an official with signing authority for their institute.

## Abbreviations

*ABCG2*: ATP-binding cassette subfamily G member 2
*β*_FS_: Full-sib regression slope
*β*_OM_
*β*_OP_: Offspring-parent regression slope
BMI: Body mass index
GLUT9: Glucose transporter 9
GWAS: Genome-wide association studies
*h*^2^: Heritability in the narrow sense
*LRP2*: Low-density lipoprotein receptor-related protein 2
NHLBI: National Heart, Lung, and Blood Institute
*PKD2*: Polycystin 2, transient receptor potential cation channel
*SLC2A9*: Glucose transporter type 9
SD: Standard deviation
SE: Standard error
SNP: Single nucleotide polymorphism.

## Supplementary Materials

Supplementary Materials Supplementary Table 1 presents the location of the SNPs presented in Discussion.
