# Supplementary material for: Quantile-Dependent Expressivity of Serum Uric Acid Concentrations
Source: Int J Genomics. 2021 Sep 2;2021:3889278. doi: 10.1155/2021/3889278 (PMC8448993; doi:10.1155/2021/3889278)
Supplement: Supplementary Materials — Supplementary Table 1 presents the location of the SNPs presented in Discussion. [file 3889278.f1.docx]

| Supplementary table 1. Table of SNPs reporting gene-environment interactions. | | | |
| --- | --- | --- | --- |
| SNP | Chromosome | Position | Gene |
| rs13120819 | 4 | 89160677 | *5' of ABCG2* |
| rs2231142 | 4 | 89052323 | *ABCG2* |
| rs2544390 | 2 | 170204846 | *LRP2* |
| rs11722228 | 4 | 9915741 | *SLC2A9* |
| rs12510549 | 4 | 10276467 | *SLC2A9* |
| rs2725220 | 4 | 88959922 | *SLC2A9* |
| rs3733591 | 4 | 9922130 | *SLC2A9* |
| rs6449213 | 4 | 9994215 | *SLC2A9* |
| rs6855911 | 4 | 9935910 | *SLC2A9* |
| rs7442295 | 4 | 9966380 | *SLC2A9* |
| Source Tseng CC, Wong MC, Liao WT, Chen CJ, Lee SC, Yen JH, Chang SJ. Systemic Investigation of Promoter-wide Methylome and Genome Variations in Gout. Int J Mol Sci. 2020;21:4702. doi: 10.3390/ijms21134702. | | | |
